# Indole-3-Carbinol Derivative DIM Mitigates Carbon Tetrachloride-Induced Acute Liver Injury in Mice by Inhibiting Inflammatory Response, Apoptosis and Regulating Oxidative Stress

**DOI:** 10.3390/ijms21062048

**Published:** 2020-03-17

**Authors:** Suvesh Munakarmi, Lokendra Chand, Hyun Beak Shin, Kyu Yun Jang, Yeon Jun Jeong

**Affiliations:** 1 Laboratory of Liver Regeneration, Biomedical Research Institute, Chonbuk National University Medical School, Jeonju 54907, Korea; sanghzain@gmail.com (S.M.); chand_lb76@yahoo.com (L.C.); 2 Department of Surgery, Chonbuk National University Hospital, Jeonju 54907, Korea; no1kal@naver.com; 3 Department of Pathology, Chonbuk National University Hospital, Jeonju 54907, Korea; kyjang@chonbuk.ac.kr

**Keywords:** DIM, carbon tetrachloride (CCl_4_), nuclear factor erythroid-2-related factor 2 (Nrf2), hemeoxygenase-1 (HO-1), inflammation, apoptosis, oxidative stress

## Abstract

3,3′-Diindolylmethane (DIM), a metabolic product of indole-3-carbinol extracted from cruciferous vegetables exhibits anti-inflammatory and anti-cancer properties. Earlier, the product has been demonstrated to possess anti-fibrotic properties; however, its protective effects on liver injury have not been clearly elucidated. In this study, we postulated the effects and molecular mechanisms of action of DIM on carbon tetrachloride (CCl_4_)-induced liver injury in mice. Acute liver injury was induced by a single intraperitoneal administration of CCl_4_ (1 ml/kg) into mice. DIM was injected via subcutaneous route for three days at various doses (2.5, 5 and 10 mg/kg) before CCl_4_ injection. Mice were sacrificed and serum was collected for quantification of serum transaminases. The liver was collected and weighed. Treatment with DIM significantly reduced serum transaminases levels (AST and ALT), tumor necrosis factor-α (TNF-α) and reactive oxygen species (ROS). CCl_4_- induced apoptosis was inhibited by DIM treatment by the reduction in the levels of cleaved caspase-3 and Bcl2 associated X protein (Bax). DIM treated mice significantly restored Cytochrome P450 2E1, nuclear factor erythroid 2-related factor 2 (Nrf2) and heme oxygenase-1 (HO-1) expression in CCl_4_ treated mice. In addition, DIM downregulated overexpression of hepatic nuclear factor kappa B (NF-κB) and inhibited CCl_4_ mediated apoptosis. Our results suggest that the protective effects of DIM against CCl_4_- induced liver injury are due to the inhibition of ROS, reduction of pro-inflammatory mediators and apoptosis.

## 1. Introduction

The liver, a vital organ, acts as an accessory digestive gland that produces biochemicals necessary for digestion, detoxifies various metabolites and synthesizes proteins. Therefore, pathogenesis in the liver rises due to the involvement of numerous cytokines and growth-factor-mediators [1]. Depending upon the characteristics, liver injuries are reversible and are mostly self-healing [2]. However, the progression of repeated injury and rapid dysfunction of liver can lead to multi-organ failure and even death [3]. Liver injury can be acute or chronic resulting from multiple reasons such as viral hepatitis, drug overdose, idiosyncratic drug reaction and toxins [4]. To date, the characteristics of liver injury have been explored extensively, but no effective therapeutic steps have been implemented [5,6].

Oxidative stress and inflammation are considered as a common pathological mechanism to be involved in the initiation and progression of liver injury [7,8]. Both enzymatic antioxidants such as superoxide dismutase (SOD) and catalase (CAT) and non-enzymatic antioxidants such reduced glutathione (GSH) are important for cellular response and are used as indexes to evaluates the level of oxidative stress [9,10,11]. Nrf2/HO-1 cascade has found to be a protective master regulator against liver disease through the means of cellular defense by mediating antioxidant response and anti-inflammatory and cytoprotective properties. Loss or dysregulation of Nrf2/HO-1 activity was found to be correlated with the development of chronic inflammatory diseases [12,13,14,15,16]. Therefore, antioxidant and anti-inflammatory therapies are proposed to prevent and treat liver injury.

Carbon tetrachloride (CCl_4_) is a well-characterized drug used to induce hepatic injury widely in scientific research [17]. CCl_4_ is believed to be involved in inducing multiple phases of liver injury. The first event of liver injury is the disruption in the permeability of plasma, lysosomes, and mitochondrial membrane [18]. Formation of highly reactive free radicals by the metabolic activity of liver enzyme Cytochrome P450 2E1 (CYP2E1) to trichloromethyl radical (CCl_3_*) leads to oxidative degradation of lipids [19]. The second event of CCl_4_ induced liver injury involves the formation of pro-inflammatory cytokines such as Tumor necrosis factor (TNF-α) that stimulate Kupffer cells, thereby resulting in the production of pro-inflammatory mediators [20]. Production of the cytokines leads to apoptosis of hepatocytes and liver inflammation [21]. The formation of inflammatory cytokines increases reactive oxygen species (ROS) generation along with the stimulation of oxidative stress with consequent advancement of liver injury [22]. Consequently, the blocking of various inflammatory pathways and inhibiting oxidative stress provides an effective aid to heal liver injury.

3,3′-diindolylmethane (DIM) is a major bioactive precursor of Indole-3-carbinol extracted from cruciferous vegetables such as broccoli, Brussels sprouts, cabbage and kale [23]. Previous studies reported that DIM has numerous preventive roles, including anti-inflammatory, free radical scavenging, anti-oxidant and anti-cancer effects [24]. Recent studies have noted the protective effect of DIM against liver injury, cardiac-inflammatory responses and renal fibrosis. S Tomar et al. reported that DIM inhibits lipopolysaccharide (LPS)-mediated liver injury by targeting Interleukin-1 Receptor-Associated Kinase 4 (IRAK4) and modulating Toll-like receptor signaling [25]. Luo et al. reported that DIM attenuates LPS-induced inflammatory responses and apoptosis in cardiomyopathy [24]. Additionally, DIM inhibited fibrosis by inhibiting TGFβ/Smad2/3 signaling pathways [26]. However, the protective effects of DIM in CCl4 induced liver injury remain unclear. Therefore, we aim to explore the potential therapeutic effects and mechanism of action of DIM in the case of CCl_4_-induced liver injury.

## 2. Results

### 2.1. DIM Inhibits CCl_4_ Induced Liver Injury

**Figure 1:**
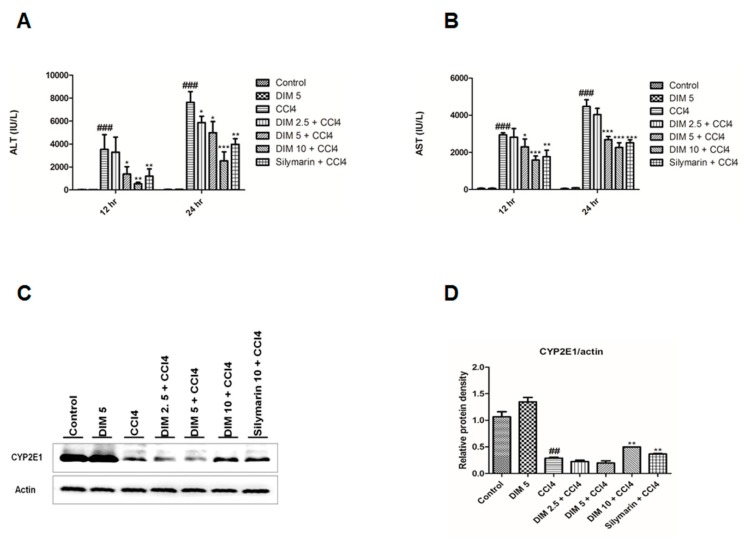
Effects of 3,3′-diindolylmethane (DIM) on serum levels of alanine aminotransferase (ALT, **A**) and aspartate aminotransferase (AST, **B**) at 12 and 24 h after CCl_4_ administration. Data are expressed as mean ± SD (*n* = 5). (**C**) Immunoblot analysis of Cytochrome P450 2E1 (CYP2E1) at 24 h after CCl_4_ injection. (**D**) Quantification of relative protein expression normalized to β-actin. Data are expressed as mean ± SD (*n* = 3). ^###^ *p* < 0.001 and ^##^ *p* < 0.01 denotes significant differences compared to the normal control group, * *p* < 0.05, ** *p* < 0.01, *** *p* < 0.001 compared to the CCl_4_ group.

Analysis of serum AST and serum ALT is an essential biochemical analysis for determining liver function. The effect of DIM on the serum AST, ALT levels and protein expression of CYP2E1 in CCl_4_-treated mice is shown in Figure 1A–C. CCl_4_ treatment gradually increased the activities of serum AST and ALT and dramatically decreased CYP2E1 expression. However, pretreatment with DIM and silymarin remarkably decreased the AST, ALT levels and restored the expression of CYP2E1 in CCl_4_-treated mice. These results illustrate that DIM significantly reverses the effects of CCl_4_ in a dose-dependent manner.

### 2.2. DIM Mitigates CCl_4_-Induced Hepatic Histopathological Damage

**Figure 2:**
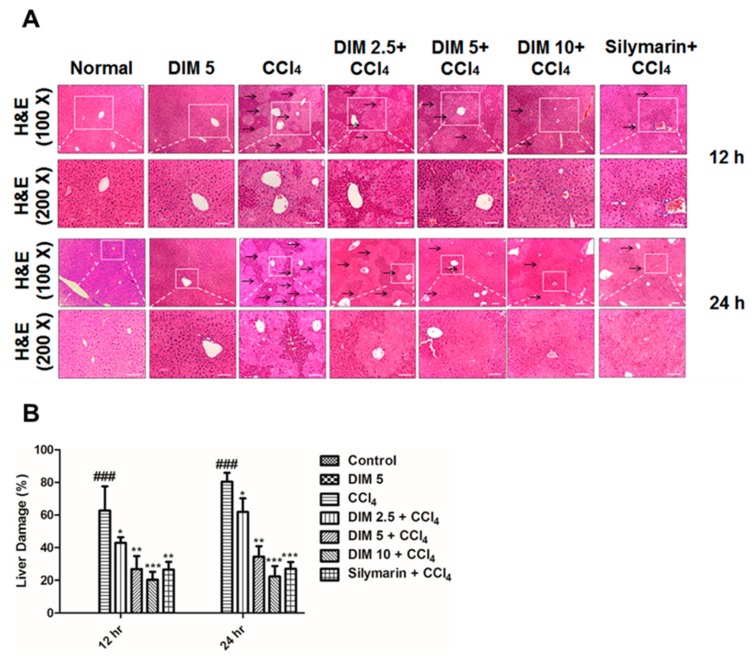
Effects of DIM on histopathological changes of liver tissues; the black arrow shows the necrotic area and liver damage (**A**) and the quantitative measurement (%) area of damage (**B**) of liver tissues after CCl_4_ injection. The tissues were stained with H&E. The liver sections were observed at X100 and X200 magnification. The scale bar represents 50 and 100 μm, respectively. Data are expressed as mean ± SD (*n* = 5). ^###^ *p* < 0.001 denotes significant differences compared to the normal control group, * *p* < 0.05, ** *p* < 0.01, *** *p* < 0.001 denotes significant difference compared to the CCl_4_ group.

Figure 2 shows the extent of histopathological damage as examined by H&E staining in liver sections. Histopathological feature of CCl_4_-induced liver injury was characterized based on shrinkage of nuclei, multiple area of portal inflammation and massive hepatocyte necrosis, which were significantly attenuated by pretreatment with DIM (2.5, 5 and 10 mg/kg) and silymarin (10 mg/kg) in a dose-dependent manner (Figure 2A,B and Figure S1).

### 2.3. DIM Pretreatment Inhibits CCl_4_-Induced Oxidative Stress and ROS Production in Response to CCl_4_ Administration

**Figure 3:**
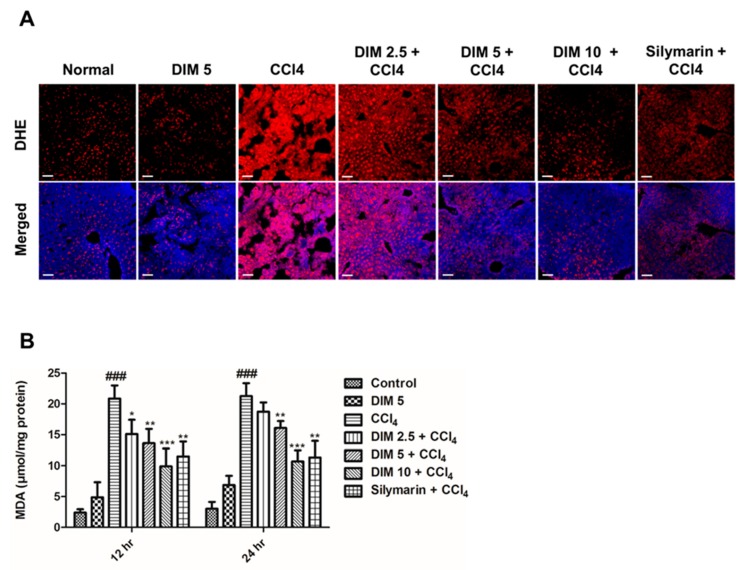
DIM pretreatment attenuates CCl_4_-induced oxidative stress and ROS production in mice. (**A**) Cryostat liver sections were treated with 5 μM (DHE) dihydroethidium at 37 °C for 30 min, washed with PBS and mounted with DAPI and assessed using a confocal microscope. The scale bar represents 30 μm. (**B**) MDA levels were measured using a commercial kit. Data are presented as mean ± SD (*n* = 5). ^###^ *p* < 0.001 determined as significant differences compared to the normal control group, * *p* < 0.05, ** *p* < 0.01, *** *p* < 0.001 compared to the CCl_4_ group.

The generation of reactive oxygen species and increased lipid peroxidation are considered as important factors for the determination of chemically induced liver injury in mice. To determine the protective effects of DIM on CCl_4_-induced oxidative stress, the intensity of ROS production and the levels of MDA, in the liver were examined as shown in Figure 3. In comparison with the control group, mice from CCl_4_ injury groups showed significantly increased intensity of red fluorescence ROS and elevated MDA levels and as shown in (Figure 3A,B). DIM pretreatment significantly attenuated the level of oxidative stress marker and MDA and lowered the DHE fluorescence, suggesting that DIM probably inhibits CCl_4_-induced hepatic damage by reducing oxidative stress and inhibiting the production of ROS in a –dose-dependent manner.

### 2.4. DIM Pre-Treatment Modulates Antioxidant Activity by Regulating the Nrf2/HO-1 Signaling Pathway and Inhibits Oxidative Stress in Response to CCl_4_ Administration

**Figure 4:**
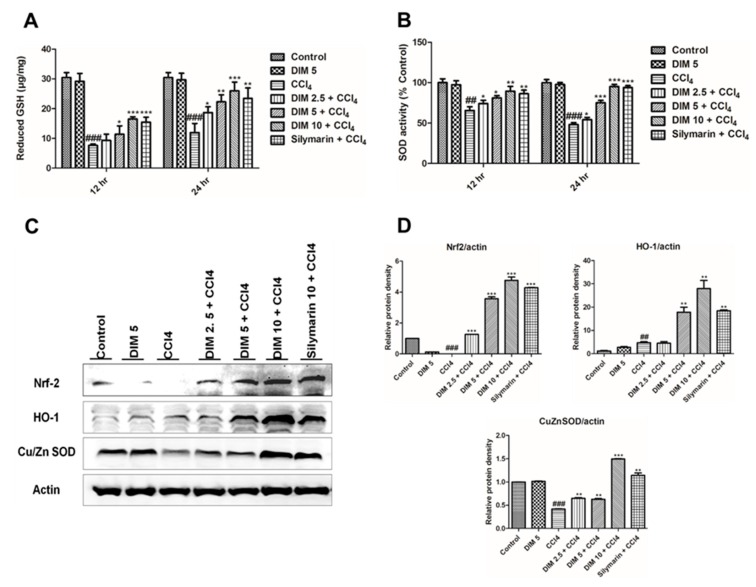
DIM increased detoxification and improved antioxidant ability by regulating the Nrf2/HO-1 signaling pathway and inhibiting oxidative stress in CCl_4_-induced liver injury in mice. (**A**) Reduced glutathione (GSH); (**B**) superoxide dismutase (SOD) activity; data are expressed as mean ± SD (*n* = 5). (**C**) Protein expression of Nrf2, HO-1, and Cu/Zn SOD at 24 h after CCl_4_ injection by using Western blot analysis. (**D**) Quantification of relative protein expression normalized to β-actin. Data are expressed as mean ± SD (*n* = 3). ^###^ *p* < 0.001 and ^##^ *p* < 0.01 denotes significant differences compared to the normal control group, * *p* < 0.05, ** *p* < 0.01, *** *p* < 0.001 compared to the CCl_4_ group.

Previous studies elucidate that the Nrf2/HO-1 signaling pathway plays an important role in CCl_4_-induced liver injury by inhibiting oxidative stress. Furthermore, to analyze the molecular mechanism underlying the protective effect of DIM against CCl_4_-induced oxidative injury, we measured the levels of reduced GSH and SOD activity as shown in Figure 4A,B, whereas protein levels of Cu/Zn SOD, Nrf2 and its target genes were measured by Western blot analysis (Figure 4C). DIM pretreatment significantly improved the levels of endogenous antioxidant such as reduced GSH and SOD and induced the protein levels of Nrf2, HO-1, and Cu/Zn SOD. These results suggest that DIM enhances the antioxidant ability through Nrf2/HO-1 activation and inhibits oxidative stress.

### 2.5. DIM Pre-Treatment Inhibits CCl_4_-Induced Inflammatory Mediators and Cytokines

**Figure 5:**
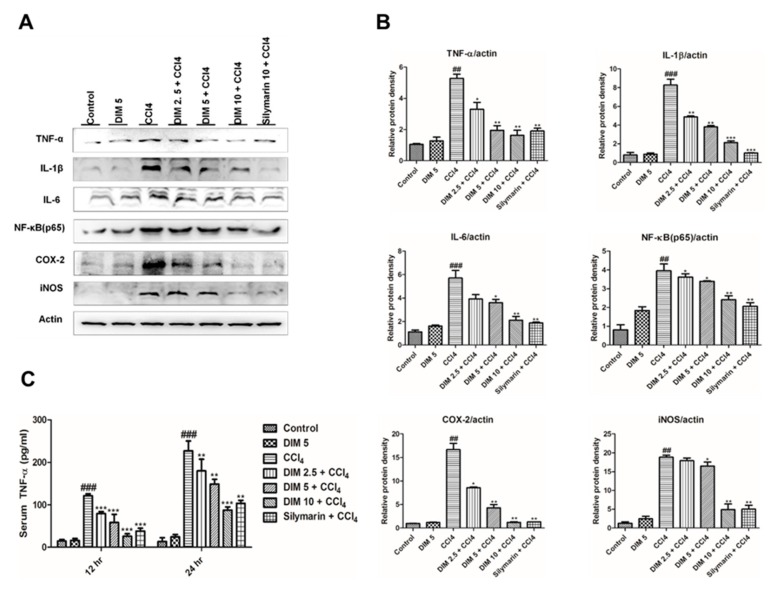
DIM pretreatment inhibits the protein expression levels of inflammatory factors in liver tissue of CCl_4_-induced liver injury in mice. (**A**) Protein expression of pro-inflammatory cytokines (TNF-α, IL-6 IL-1β) and inflammatory mediators (COX-2 and iNOS) at 24 h after CCl_4_ injection by using Western blot analysis. (**B**) Quantification of relative protein expression normalized to β-actin. (**C**) The level of serum TNF-α measured using ELISA kits. The values represent the mean ± SD (*n* = 3). ^###^ *p* < 0.001 and ^##^ *p* < 0.01 denotes significant differences compared to the normal control group, * *p* < 0.05, ** *p* < 0.01, *** *p* < 0.001 compared to the CCl_4_ group.

Inflammatory response plays a key role in the progression of liver damage. To reveal the mechanisms by which DIM inhibits inflammatory response induced by CCl_4,_ Western blot analysis, and enzymatic assay were performed. As shown in Figure 5, the protein expression of three important inflammatory cytokines (TNF-α, IL-6, IL-1β) and serum TNF-α were significantly increased in the CCl_4_ group. However, pretreatment of DIM inhibited these elevations. Cyclooxygenase-2 (COX-2) and inducible nitric oxide synthase (iNOS) are two important enzymes known as key executors of uncontrolled inflammation by producing prostaglandin and NO, respectively. Pretreatment of DIM significantly suppressed hepatic COX-2 and iNOS expression levels induced by CCl_4_ administration. These findings suggest that DIM protects the liver from injury by inhibiting the production of inflammatory cytokines and mediators.

### 2.6. DIM Pre-Treatments Attenuates CCl_4_-Induced Hepatocyte Apoptosis in Mice

**Figure 6:**
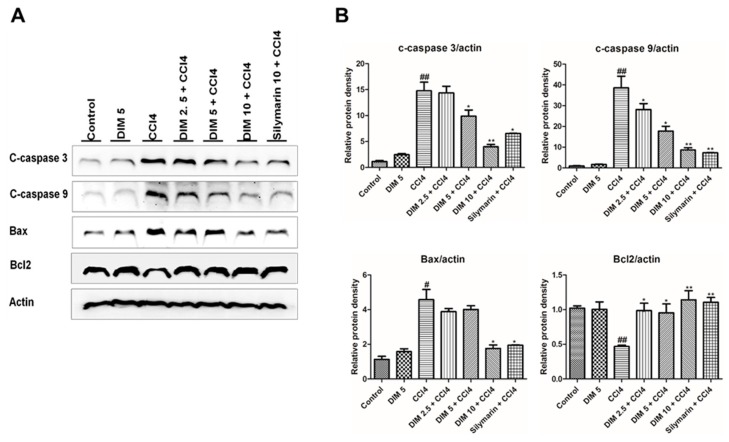
Pretreatment of DIM inhibits CCl_4_-induced hepatic cell death. (**A**) Western blot analysis showing the expression of apoptotic proteins (cleaved caspase-3, cleaved caspase-9); pro-apoptotic proteins Bax and anti-apoptotic protein Bcl2 at 24 h after CCl_4_ injection. (**B**) Quantification of relative protein expression normalized to β-actin. The values represent the mean ± SD (*n* = 3). ^#^ *p* < 0.05 and ^##^ *p* < 0.01 denoted significant differences compared to the normal control group, * *p* < 0.05, ** *p* < 0.01, *** *p* < 0.001 compared to the CCl_4_ group.

In order to determine the hepatoprotective effect of DIM against CCl_4_-induced apoptosis, the levels of various apoptotic and anti-apoptotic markers were analyzed by Western blot. As shown in Figure 6, CCl_4_ administration significantly decreases the expression of the anti-apoptotic protein, Bcl2, while increasing the expression of the pro-apoptotic protein, Bax. However, these expressions were reversed with DIM pretreatment. Activation of caspase plays an important role in apoptosis; CCl_4_ administration remarkably increased the cleavage of caspase-3 and caspase-9, suggesting severe apoptosis, while these elevations were attenuated by pretreatment with DIM. These results suggest that DIM imparts a protective effect against CCl_4_-induced liver injury by suppressing apoptotic response.

## 3. Discussion

The Liver is one of the important vital organs that helps in maintaining various metabolic activities and functions of the body. Any injury to the liver leads to oxidative stress and inflammation [27]. CCl_4_-induced liver injury is the most commonly used experimental model to evaluate the hepato-protective effect of natural products [28]. There are many side effects of Western medicines that have been reported for the treatment of liver injury [29]. Therefore, natural medicines have become a future potential therapeutic hope for controlling liver disease. Although several studies have reported anti-inflammatory, anti-cancer and antioxidant effects of DIM [26,30], the molecular mechanism and regulation of DIM in the suppression of inflammatory response, inhibition ROS-induced lipid peroxidation and apoptosis in CCl_4_-induced liver injury remain unknown. In the present study, we attempt to determine the antioxidative, anti-inflammatory and apoptotic effects of DIM in CCl_4_ – induced liver injury.

Elevated levels of serum AST and serum ALT are the major indices of CCl_4_-induced liver toxicity with damage in the cell membrane and loss of functional integrity of hepatocytes [31]. The toxicity of CCl_4_ often results in the formation of free radical trichloromethyl in the liver by cytochrome P450 enzyme (CYP2E1) in the endoplasmic reticulum of hepatocytes thereby causing severe liver damage [32]. However, some studies found that CCl_4_ inhibits the expression of CYP2E1 due to the labilization and inactivation caused by ongoing oxidative and apoptosis [33,34,35]. Previous studies stated that CCl_4_ dramatically increased the serum ALT and AST levels and led to changes in the membrane integrity of hepatocytes in mice [36,37]. In the current study, pretreatment of DIM significantly lowered the levels of liver enzymes (parameters of liver injury) and potentially stabilized the hepatic histological changes by decreasing hepatic damage in dose and time-dependent manner, suggesting that DIM may serve as a novel approach for the treatment of CCl_4_-induced liver injury in mice.

The end product of lipid peroxidation is MDA, which is considered as an indicator of ROS for measuring oxidative stress induced by CCl_4_ injury. [38,39]. In order to validate the hepatoprotective and antioxidant ability of DIM, endogenous levels of antioxidant enzymes such as superoxide dismutase (SOD) and reduced glutathione (GSH) were measured. SOD and reduced GSH are the first lines of the antioxidative defense system. SOD is important for the conversion of superoxide radicals into H_2_O_2_ and O_2_ whereas reduced GSH is important in catalyzing the reduction of hydrogen peroxide and preventing them from the free radical formation. [40,41]. The findings of this research are consistent with previous studies that show ROS after CCl_4_ treatment inactivated the antioxidant enzymes SOD and reduced GSH [42]. Our data showed that DIM effectively decreased reduced GSH and restored enzymes SOD and also attenuated MDA levels in time and dose-dependent manner.

Nrf2 is a known redox-responsive transcription factor that plays an important role in regulating antioxidant response elements (AREs) and thereby regulates the expression of a battery of genes, such as HO-1, GST, GCLC and GCLM [43,44,45]. Previously, numerous studies have proven the importance of antioxidants against hepatic injury by triggering the Nrf2/HO-1 signaling pathway [46,47]. Quite a few studies have reported that Nrf2 was involved in protection against CCl_4-_induced liver injury [48,49]. Nrf2 plays a key role in regulating the antioxidant defense system in response to CCl_4_ induced oxidative stress, and activation of Nrf2 is considered as a strong antioxidant and protection against liver injury [50,51]. Consistent with other studies, we found that CCl_4_ noticeably decreased protein levels of Nrf2 and increased HO-1 expression, while DIM pretreatment restored the expression of these proteins thereby indicating that DIM exhibits protective against CCl_4-_induced liver injury by activating Nrf2/HO-1 signaling pathway.

Elicitation of inflammation response plays a crucial role in the pathological process of CCl_4_-induced liver injury [52,53]. Previous studies have shown that the expression of pro-inflammatory cytokines (TNF-α, IL-6, IL-1β) and inflammatory mediators (COX-2 and iNOS) plays important role in the development and maintenance of inflammation related to liver injury [19,54,55,56]. iNOS is responsible for the production NO, which is a highly reactive molecule synthesized from L-arginine. Overproduction of NO is one of the causes of inflammatory responses by inhibiting the growth of lymphocytes and damaging surrounding cells and tissues [53,57]. In this study, the expression of IL-6, IL-1β, COX-2, iNOS, and TNF-α in both the serum and liver were substantially increased by CCl_4_ thereby leading to liver damage, which was inhibited by DIM pre-treatment. These results suggested that DIM exhibits an anti-inflammatory effect by suppressing the inflammatory response induced by CCl_4_.

Apoptosis and necrosis play an important role to contribute in the progression of liver injury [58,59], but it is still unclear whether necrosis or apoptosis is dominant in CCl_4_-induced liver injury [60]. The previous study revealed that CCl_4_-induced acute liver damage is characterized by necrotic cell death [58], while another study suggested the involvement of CCl_4_-induced hepatic cell apoptosis [60]. In our study, the CCl_4_-induced hepatic injury model followed the apoptotic pathway where caspase activation was involved. Several pro-apoptotic and anti-apoptotic proteins such as Bax and Bcl2 are responsible for cellular apoptosis [61,62,63,64]. Bax is an important pro-apoptotic gene in the Bcl2 family, which may translocate to the mitochondria to induce apoptosis [65], whereas Bcl2 is an anti-apoptotic protein that suppresses apoptosis [66,67]. In our study, we found that CCl_4_ administration induced apoptosis of liver cells by significant elevation of cleaved caspase-3, cleaved caspase-9 and Bax expression while Bcl2 expression was inhibited, and these conditions were reversed in DIM pretreatment condition. These results reveal the protective effect of DIM involving alleviation CCl_4_-induced liver injury by inhibition of apoptosis.

## 4. Materials and Methods

### 4.1. Chemical and Reagents

DIM (purity > 98%) and CCl_4_ were purchased from Sigma-Aldrich Chemical Co., St. Louis, MO, USA. serum alanine transaminase (ALT) and aspartate transaminase (AST) assay kits were purchased from Asam Pharm. Co. Ltd., South Korea. TNF-α Elisa kits were purchased from Koma Biotech (Seoul, Korea). Glutathione (GSH) and malondialdehyde (MDA) were purchased from Biovision (S Militas Blvd., CV, USA) and Cell Biolabs (San Diego, CA, USA) respectively. DHE (dihydroethidium) was purchased from Invitrogen (Carlsbad, CA, USA).

### 4.2. Experimental Animals

Specific pathogen-free male FVB mice (6–8 weeks old) were purchased from Koatech (Pyeongtake, Korea). All mice were housed and given libitum access to food and water. All experimental procedures were conducted according to the ethical guidelines, and the protocols were approved by the Institutional Animal Care and Use Committee of Chonbuk National University, Jeonju, South Korea (Approved no: CBNU 2019-030).

### 4.3. Animal Model and Treatment with DIM

**Figure 7:**
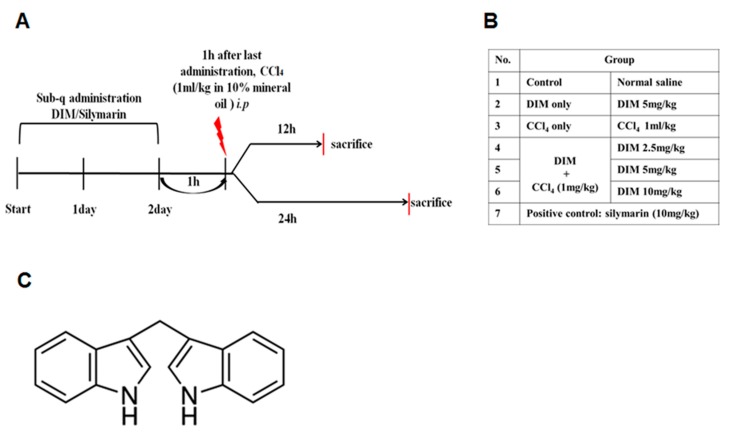
An experimental method for CCl_4_-induced liver injury model and treatment method. (**A**) diagram shows sacrifice after 12 and 24 h, (**B**) table showing treatment doses and groups, (**C**) The molecular structure of DIM.4.4. Determination of Enzymatic assay.

All mice were randomly divided into 12 and 24 h sets with seven groups of 5 mice in each set. Group I (control group) and group III (negative control) mice were injected with normal saline for 3 consecutive days. Group II (DIM only group) mice were treated with 5 mg/kg DIM, and group VII (positive control group) were subcutaneously (sub-q) treated with silymarin (10 mg/kg) for three days. Group IV-VI (DIM + CCl_4_ group) mice received (sub-q) 2.5, 5 and 10 mg/kg of DIM dissolved in PBS for three consecutive days. On the third day, 1 h after the last administration, all groups except groups I and II were injected with a single dose of CCl_4_ (10% in mineral oil), whereas animals in groups I and II were *i.p.* injected with mineral oil. Mice from each group were anesthetized and sacrificed at 12 and 24 h of CCl_4_ injection as shown in Figure 7, and samples of blood and liver tissues were collected for further analysis.

The blood samples were collected and incubated at room temperature for 30 min and centrifuged at 3000 rpm for 15 min at room temperature to separate serum. The serum transaminases (AST and ALT) levels were quantified spectrophotometrically according to the Reitman–Frankel method.

### 4.4. Determination of Enzymatic Assay

The blood samples were collected and incubated at room temperature for 30 min and centrifuged at 3000 rpm for 15 min at room temperature to separate serum. The serum transaminases (AST and ALT) levels were quantified spectrophotometrically according to the Reitman–Frankel method.

### 4.5. Measurements of Reduced Glutathione (GSH) and Superoxide Dismutase (SOD) Levels in Liver Tissue

Liver tissues were homogenized, centrifuged at 12,000 rpm for 15 min at 4 °C, and the supernatant was collected to measure the levels of reduced GSH and SOD according to the instruction given by the manufacturer (Biovision Incorp., Milpitas, CA, USA). The reduced GSH and SOD were measured spectrophotometrically at 405 and 450 nm, respectively.

### 4.6. Measurements of Malondialdehyde (MDA)

MDA levels in liver tissue homogenates were determined using a commercially available MDA assay Kit (Cell Biolabs, Inc., San Diego, CA, USA), absorbance of the colored complex was measured at a wavelength of 532 nm by kinetic spectrophotometric. The principle of the assay depends on a colorimetric determination of pink pigment product, derived from the breakdown of polyunsaturated fatty acid TBA (thiobarbituric acid).

### 4.7. Measurement of Serum TNF-α

Blood samples were collected and serum was separated at different time intervals after CCl_4_ injury to the mice. TNF-α levels in the serum were measured by using mouse TNF-α enzyme-linked immunosorbent assay kit (Koma Biotech, Inc., Seoul, Korea), according to the manufacturer’s instruction.

### 4.8. Liver Histopathology Examination

Liver tissues were collected and fixed with 4% paraformaldehyde and embedded in paraffin. Sectioned tissues measuring 5 μm in thickness were deparaffinized with xylene and stained with hematoxylin and eosin (H&E). The histological changes were observed under a light microscope at 100× and 200× magnification. Live images were captured and the area of necrosis foci was measured in randomly chosen five areas using image analysis software (Leica Application Suite Version 3.6, Leica Microsystem, Heerbrugg, Switzerland).

### 4.9. Microscopic Detection of Reactive Oxygen Species

Cryosections from snap-frozen liver (5μm) tissues were prepared. In situ ROS detection was performed as described by Lehwald et al., [68] using dihydroethidium (DHE; Invitrogen, Carlsbad, CA, USA). Briefly, cryosections were stained with 5 μM DHE for 30 min and dyed for nucleus by DAPI, mounted by anti-fluorescence quenching sealing tablets in dark at room temperature and observed under a fluorescence microscope (Axioskop 2 Plus, Carls Zeiss, Gottingen, Germany).

### 4.10. Western Blot Analysis

Liver tissues were homogenized with lysis buffer (Intone Biotechnology, Seoul, Korea) supplemented with phosphatase-1 inhibitor cocktail (Sigma, St. Louis, MO, USA) on ice. Tissue homogenates were centrifuged at 13,000 rpm for 30 min at 4 °C, and supernatants were collected for Western blot analysis. Protein concentration was quantified with Bradford assay (Bio-Rad, Hercules, CA, USA), and proteins were denatured by heating at 95 °C. The denatured proteins were subjected to SDS-PAGE and transferred to the PVDF membrane (Bio-Rad, Hercules, CA, USA). After blocking the membrane with 5% skimmed milk for 1 h at room temperature, the blot was incubated either overnight at 4 °C or 1 h at room temperature with primary antibodies for anti-mouse actin (1:3000, Sigma), anti-rabbit caspase-3, caspase-9, TNF-α, COX-2 (1:1000, Cell Signaling, Danvers, MA, USA), anti-rabbit iNOS, Copper, zinc superoxide dismutase (Cu/Zn SOD), HO-1, CYP2E1 (1:1500, Enzo Life Science, Inc., USA), anti-rabbit Bax, Interleukin-1β (IL-1β), Interleukin-6 (IL-6), NF-κB (p65) and NRF2 (1:1000, Santa Cruz, Dallas, TX, USA), anti-rabbit B-cell lymphoma 2 (Bcl2) (1:1000, Bioworld Tech. Inc., Minneapolis, MN, USA). The membranes were washed and incubated with HRP-conjugated goat anti-mouse or anti-rabbit secondary antibodies. Protein expression was detected using a chemiluminescent detection kit (Millipore Corp., Billercia, MA, USA).

### 4.11. Statistical Analysis

All the experimental data are shown as the means ± standard deviation (SD). Statistical significance of differences at different time duration (12 and 24 h) was calculated with one-way ANOVA followed by unpaired student’s test using Prism 7 software (GraphPad Software, San Diego, CA, USA). The *p* values < 0.05, 0.01, 0.001 were considered to be statistically significant.

## 5. Conclusions

**Figure 8:**
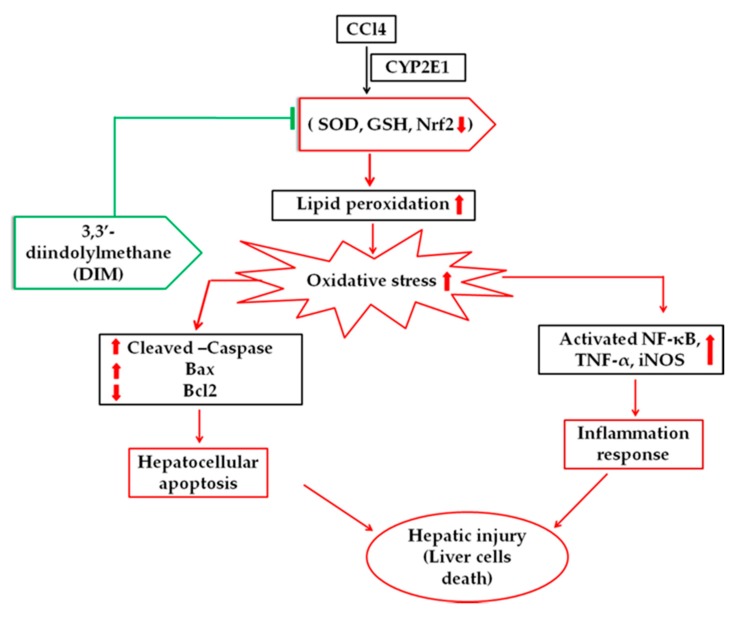
A schematic diagram of the proposed mechanisms by which DIM inhibits CCl_4_–induced oxidative stress, reduces inflammatory response and inhibits apoptosis by regulating oxidative stress and caspase-3,9/Bax/Bcl2 pathways. In the figure downward red arrow shows CCl_4_ – induced liver injury decreases antioxidants ability (SOD, GSH, Nrf2) and anti-apoptotic proteins levels Bcl2 whereas upward red arrow shows increased inflammatory responses, oxidative stress and apoptotic proteins, which were altered by DIM pretreatment.

Based on results, it was apparent that CCl_4_ administration induced liver damage by disrupting the morphology of the liver, increasing the serum ALT and AST levels, and inducing inflammatory response and oxidative stress promoting apoptosis. DIM has shown antioxidative, anti-inflammatory and anti-apoptotic abilities, reduced serum ALT and AST thereby ameliorate CCl_4_–induced liver injury by inhibiting oxidative stress (Figure 8). These outcomes suggest that DIM possesses therapeutic potential against CCl_4_–induced liver injury.

## Supplementary Materials

Supplementary materials can be found at https://www.mdpi.com/1422-0067/21/6/2048/s1. Figure S1: Effects of DIM on histopathological changes of liver tissues, showing cellular infiltration.

## Abbreviations

DIM	3,3′-Diindolylmethane
CCl_4_	carbon tetrachloride
ROS	reactive oxygen species
TNF-α	tumor necrosis factor-α
CYP2E1	cytochrome P450 2E1
Nrf2	nuclear factor erythroid 2-related factor 2
NF-κB	nuclear factor kappa B
HO-1	heme oxygenase
AST	aspartate aminotransferase
ALT	alanine aminotransferase
GSH	Glutathione
MDA	Malondialdehyde
DHE	Dihydroethidium
SOD	Superoxide dismutase
Cu/ZnSOD	Copper, zinc superoxide dismutase
COX-2	Cyclooxygenase-2
iNOS	Inducible nitric oxide synthase
IL-6	Interleukin-6
IL-1β	Interleukin-1β
Bcl2	B-cell lymphoma 2
Bax	Bcl2 associated X protein
