# Supplementary material for: Indole-3-Carbinol Derivative DIM Mitigates Carbon Tetrachloride-Induced Acute Liver Injury in Mice by Inhibiting Inflammatory Response, Apoptosis and Regulating Oxidative Stress
Source: Int J Mol Sci. 2020 Mar 17;21(6):2048. doi: 10.3390/ijms21062048 (PMC7139345; doi:10.3390/ijms21062048)
Supplement: Supplementary file 1 [file ijms-21-02048-s001.pdf]

Supplementary Figure S1.

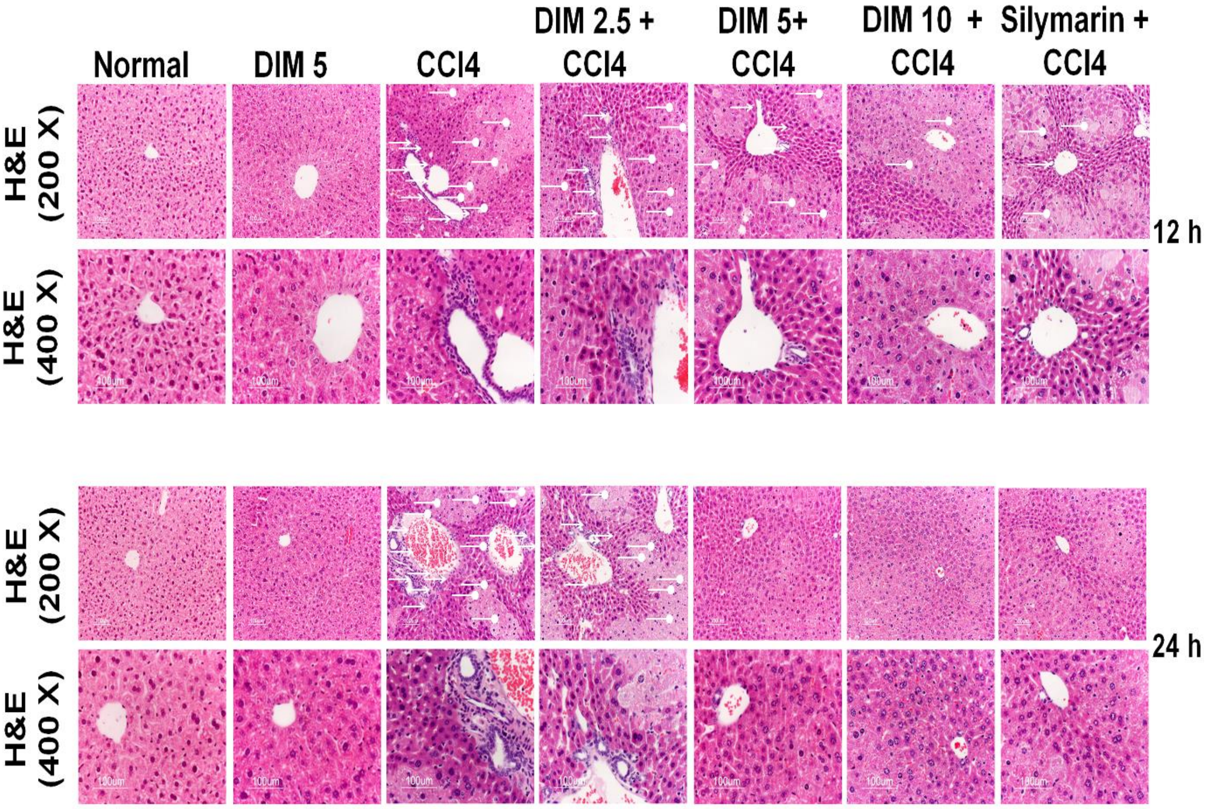

Supplementary figure S1. Effects of DIM on histopathological changes of liver tissues. The white arrows denote cellular infiltration whereas white arrow with oval head denote massive liver damage and shrinkage of nucleus. The tissues were stained with H&E. The liver sections were observed at X200 and X400 magnification.
